# Bacterial Gastrointestinal Infections in Pediatric Inflammatory Bowel Disease (PIBD)—A Single-Center Experience of Epidemiology, Management, and Outcome

**DOI:** 10.3390/diagnostics16091411

**Published:** 2026-05-06

**Authors:** Raffaela Miriam Planka, Almuthe Christine Hauer, Sebastian Bauchinger, Benno Kohlmaier

**Affiliations:** 1 Division of General Pediatrics, Department of Pediatrics and Adolescent Medicine, Medical University of Graz, 8036 Graz, Austria; raffaela.planka@stud.medunigraz.at (R.M.P.); sebastian.bauchinger@medunigraz.at (S.B.); 2 Department of Pediatrics and Adolescent Medicine, Medical University of Graz, 8036 Graz, Austria; almuthe.hauer@medunigraz.at

**Keywords:** pediatric inflammatory bowel disease, bacterial gastrointestinal infections, *Clostridioides difficile*, immunosuppressive therapy, infection management

## Abstract

**Background:** Due to dysbiosis, intestinal barrier dysfunction, and immunosuppressive therapy, pediatric inflammatory bowel disease (PIBD) patients are more susceptible to infections. However, data on bacterial gastrointestinal (GI) infections in this population are scarce, and no guidelines explicitly address immunosuppressive therapy management during such infections. This single-center study aims to address these knowledge gaps. **Methods:** A retrospective study of bacterial GI infections was conducted in PIBD patients aged 0–18 years, treated between 2011 and 2021 at the Department of Pediatrics and Adolescent Medicine, Medical University of Graz. Data to assess the study endpoints were extracted from the hospital information system. **Results:** A total of 139 PIBD patients were screened for bacterial GI infections. The mean follow-up time was 49 months (standard deviation ±33) and the total follow-up time amounted to approximately 473 person-years. Fourteen patients developed infections, with three experiencing them twice, resulting in 17 cases of infection. Most infections were caused by opportunistic bacteria, and 10 infections were treated with antibiotics (11 antibiotic prescriptions in total). At infection onset, 12 patients were on (combined) immunosuppressive therapy, including corticosteroids (3 patients), immunomodulators (9 patients), and/or biologics (3 patients). Six infections required escalation of immunosuppressive therapy due to increased PIBD activity. Hospitalization was required in five cases, and one *Clostridioides difficile* infection progressed to sepsis, necessitating intensive care unit admission. This corresponds to an incidence of three infections (95% confidence interval 1.75–4.80) and 0.2 severe infections per 100 person-years (95% confidence interval 0.01–1.11). **Conclusions:** The incidence of bacterial GI infections was 3 per 100 person-years (95% confidence interval: 1.75–4.80), with most cases being clinically mild. *Clostridioides difficile* was the most common pathogen. Immunosuppressive therapy was generally continued or intensified, when necessary, while antibiotic therapy was administered as indicated.

## 1. Introduction

Gastrointestinal (GI) infections frequently occur in pediatric populations [1]. Patients with pediatric inflammatory bowel disease (PIBD) are particularly vulnerable due to disease-related factors. GI dysbiosis, intestinal barrier dysfunction, and a higher propensity for disease severity not only increase the risk of GI infections but may also complicate the course and management of inflammatory bowel disease (IBD) [2,3,4,5]. This is often reflected in the need for intensified immunosuppressive therapy (IST), which itself is a risk factor for (opportunistic) infections.

The combination of immunosuppressive agents has become the standard treatment for moderate-to-severe IBD to enhance therapeutic efficacy [6]. However, combined IST particularly increases the risk of infections with both opportunistic and obligate pathogens [7]. A position paper by the ESPGHAN IBD Porto Group addressing infection risk and prevention in PIBD was published in 2012 and primarily refers to earlier ECCO guidelines from 2009, which were developed for adult IBD populations and have since been partially extrapolated to pediatric care [6,8]. The paper emphasizes the substantial infection risk for immunosuppressed PIBD patients, reporting bacterial infections caused by *Salmonella* species, *Listeria monocytogenes*, *Nocardia* species, and *Clostridioides difficile* (*C. difficile*). It highlights infliximab (IFX) as a particularly high-risk therapy with reported serious infections in PIBD patients [6]. In contrast, updated ECCO guidelines emphasize that the overall risk of opportunistic infections is driven primarily by corticosteroids and combined IST, with observational data still suggesting an elevated risk for anti-TNF agents such as infliximab [7].

Further evidence comes from the REACH study, which indicates that shorter IFX infusion intervals (every 8 weeks vs. 12) increase infection rates in pediatric Crohn’s disease (CD), despite improving therapeutic efficacy [9].

Several factors should be considered when deciding whether to use antibiotics for treating bacterial GI infections. Guidelines on acute infectious gastroenteritis in pediatric patients recommend that antibiotic therapy be guided by the patient’s age, the causative pathogen—since some bacteria require eradication only in immunocompromised individuals—and the clinical course of the infection [10]. PIBD guidelines [11,12,13] provide no specific recommendations on antibiotic therapy or IST management during bacterial GI infections. The ECCO guideline on opportunistic infections in IBD patients offers more detailed antibiotic therapy guidance and recommends discontinuing IST during infection until resolution [7].

Although research has focused on specific enteric pathogens associated with IBD flares, particularly *C. difficile*, studies examining the broader spectrum of bacterial species and their distribution in relation to bacterial GI infections and disease exacerbations in PIBD patients remain limited [14].

The aim of this study was to characterize the epidemiology, clinical presentation, management, and outcomes of bacterial GI infections in PIBD in a real-world cohort.

The primary objective focused on describing the epidemiology and clinical features of these infections. Secondary objectives included evaluating management strategies, particularly immunosuppressive and antibiotic therapy, as well as clinical outcomes.

## 2. Materials and Methods

This single-center, retrospective study was conducted at the Department of Pediatrics and Adolescent Medicine, Medical University of Graz. PIBD patients aged 0–18 years were identified through the hospital’s information system (MEDOCS^®^, version 7.7) and screened for bacterial GI infections. Eligible patients had a histologically confirmed PIBD, were treated at the center between 2011 and 2021, and had at least one bacterial GI infection confirmed by pathogen detection in stool or blood. For *C. difficile* infections, only toxigenic strains were considered. Infections were defined by the presence of clinical symptoms and/or elevated inflammatory markers. No specific exclusion criteria were defined.

Microbiological testing was performed based on clinical symptoms. Stool diagnostics were initiated in patients presenting with new GI symptoms suggestive of infectious enteritis, including increased stool frequency, abdominal pain, hematochezia, or unexplained elevations in inflammatory markers. Standard institutional diagnostic procedures were applied throughout the study period, including stool cultures for enteric bacterial pathogens and testing for toxigenic *C. difficile* according to hospital laboratory protocols. Detection of toxigenic *C. difficile* was required for classification as CDI, and results were interpreted in conjunction with clinical findings to minimize misclassification as asymptomatic colonization. Recurrent infection was defined as a new episode of microbiologically confirmed bacterial GI infection occurring after documented clinical resolution of a previous episode.

Data extracted from MEDOCS were used to assess primary endpoints, including infection management, IST at infection onset, IST adjustments during infection, and antibiotic use. Secondary endpoints encompassed demographic and disease-related characteristics (age, sex, PIBD type and phenotype, age at diagnosis, and interval from PIBD diagnosis to infection as well as laboratory markers of inflammation, including complete blood count, albumin, C-reactive protein, erythrocyte sedimentation rate, procalcitonin, and fecal calprotectin). Clinical severity indicators, including length of hospital stay and need for intensive care, were also documented. These secondary endpoints provided a comprehensive clinical and laboratory framework to characterize infection episodes in the PIBD population.

Incidence rates were calculated as the number of infection events per total person-years of follow-up and are reported per 100 person-years. Ninety-five percent confidence intervals were estimated assuming a Poisson distribution.

This study was conducted in accordance with the STROBE (Strengthening the Reporting of Observational Studies in Epidemiology) guidelines.

## 3. Results

A total of 139 PIBD patients were screened for bacterial GI infections. Fourteen patients experienced 17 infection episodes, corresponding to an incidence of three infections per 100 person-years (95% CI: 1.75–4.80). The median age at infection was 14 years (IQR 13–17), and the median interval from PIBD diagnosis to infection onset was 17 months (IQR 10–28). Eight patients had Crohn’s disease (CD) and six had ulcerative colitis (UC). CD phenotypes were predominantly colonic or ileocolonic, with upper GI tract involvement in all CD patients. Among UC patients, five of six initially presented with pancolitis, and two also exhibited upper GI tract involvement.

*C. difficile* was the most frequently detected pathogen, accounting for 12 of 17 infections, consistent with the known burden of CDI in PIBD patients [15,16,17]. *Klebsiella oxytoca* (*K. oxytoca*) was identified twice, while *Campylobacter jejuni* (*C. jejuni*), *Salmonella Enteritidis* (*S. Enteritidis*), *Arcobacter butzleri*, and *Aeromonas hydrophila* were each detected once. One infection represented a co-infection with *C. difficile* and *S. Enteritidis*. Three patients experienced recurrent infections, all of which were caused exclusively by *C. difficile*. Clinical presentation was dominated by diarrhea (12/17), abdominal pain (9/17), and blood or mucus in the stool (9/17), while nausea or vomiting was less common (5/17). Laboratory values obtained closest to the infection episodes showed elevated inflammatory markers (C-reactive protein) in 13/17 (76%) episodes.

At infection onset, 12 of 14 patients were receiving IST, including azathioprine, corticosteroids, methotrexate, tacrolimus, and/or infliximab, alone or in combination. During seven infection episodes, IST was intensified or newly initiated, most often by increasing corticosteroid doses or adding azathioprine. Immunosuppression was discontinued in only one case, due to drug intolerance rather than the infection itself.

Antibiotics were prescribed in 11 of 17 episodes. In one episode, the prescribed therapy was not taken. Most *C. difficile* infections (*n* = 12) were treated with metronidazole (8/12, 67%) or vancomycin (1/12, 8%), consistent with guideline recommendations [7,10]. *K. oxytoca* infections (*n* = 2) were treated with ciprofloxacin or empirically with metronidazole. Infections caused by other pathogens received either pathogen-directed or empirically initiated antibiotics, and prior antibiotic therapy at another institution accounted for one undocumented case. Antibiotic therapy did not lead to IST discontinuation in any patient.

Hospitalization was required for five infections, with a median hospital stay of four days (range 2–13). Two cases were caused by *C. difficile*, two by *K. oxytoca*, and one by *C. jejuni*. One patient with CDI developed sepsis and required intensive care, corresponding to 0.2 severe infections per 100 person-years (95% CI: 0.01–1.11). 

**Figure 1:**
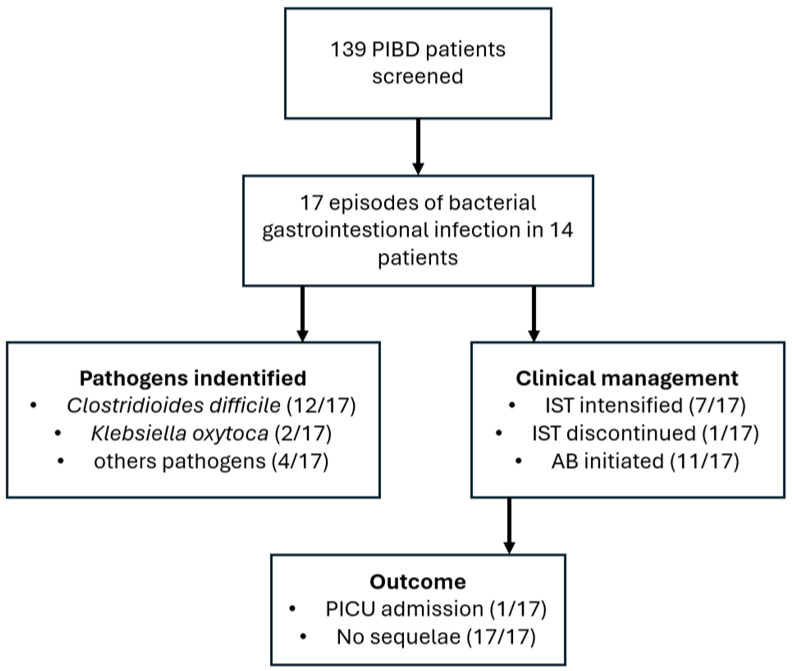
Study flowchart and clinical course of bacterial gastrointestinal infections in PIBD. Abbreviations: PIBD—pediatric inflammatory bowel disease; IST—immunosuppressive therapy; AB—antibiotics; PICU—pediatric intensive care unit.

**Figure 2:**
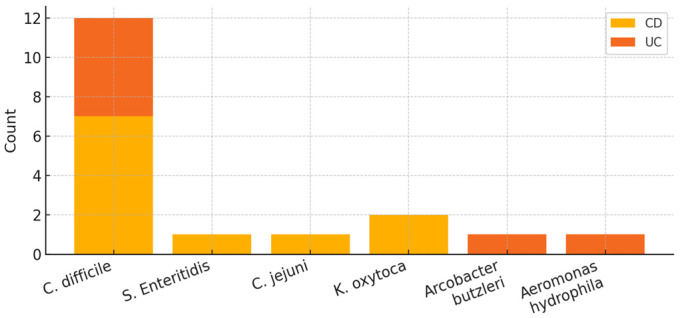
Distribution of pathogens by PIBD diagnosis (stacked bar chart). Abbreviations: PIBD—pediatric inflammatory bowel disease; CD—Crohn’s disease; UC—ulcerative colitis; *C. difficile*—*Clostridioides difficile*; *S. Enteritidis*—*Salmonella Enteritidis*; *C. jejuni*—*Campylobacter jejuni*; *K. oxytoca*—*Klebsiella oxytoca*.

The main findings are summarized in the study flowchart (Figure 1), illustrating the cohort, clinical course, and management. The distribution of pathogens is shown in Figure 2. Table 1 presents patient level data. For each patient, PIBD type and phenotype, age at PIBD diagnosis and at infection, causative pathogen(s) of the infectious episode, and immunosuppressive and antibiotic treatment at the time of and during infection are reported. Laboratory results obtained closest to the patients’ outpatient visits or hospitalizations for infection episodes are presented in Table 2.

## 4. Discussion

In this 10-year, single-center cohort of PIBD patients, bacterial GI infections occurred at a rate of 3 per 100 person-years in our cohort, with severe cases being rare. Most infections were mild and predominantly caused by C. difficile, consistent with the known susceptibility of PIBD patients to this pathogen and with epidemiological data from pediatric cohorts [15,16,18,19]. The observed proportion of bacterial GI infections in our cohort is comparable to previously reported rates in PIBD populations, although direct comparison is limited by differences in study design and diagnostic strategies. The pathogen distribution in our cohort mirrors previous studies, which identified C. difficile, Salmonella spp. and Campylobacter spp. as common enteric triggers of GI symptoms and IBD flares in PIBD patients [6,7,14].

Evidence from previous IBD studies indicates that infection risk is increased in patients treated with infliximab, particularly at shortened dosing intervals, and in those receiving corticosteroids [7,9]. In line with this, 82% of patients in our cohort were receiving IST at infection onset, often in combination regimens, reflecting the known association between intensified IBD treatment and infection susceptibility. Despite this increased risk, current PIBD guidelines provide limited recommendations on managing IST during active bacterial infections [11,12,13]. This lack of guidance creates a clinical dilemma, as enteric infections in PIBD patients are frequently accompanied by increased disease activity or flare-like symptoms. In our cohort, IST was discontinued during infection in one episode (6%) and was continued or escalated in 7 episodes (41%) in response to worsening PIBD activity. This management strategy reflects real-world practice, where maintaining or restoring disease control is prioritized to prevent uncontrolled PIBD flares, even in the presence of a concurrent infection.

Our findings highlight the clinical significance of *C. difficile* in PIBD. It accounted for 12/17 (71%) infections, contributed to most cases requiring IST escalation, and was responsible for the only severe infection resulting in sepsis and ICU admission. These results are consistent with previous reports indicating an increased risk of CDI in PIBD patients compared with both non-IBD children and adults with IBD [18,19].

Furthermore, CDI has been associated with active colonic inflammation, increased healthcare utilization, and more severe disease courses in PIBD [20,21]. The predominance and clinical impact of CDI in our cohort underscore the need for prompt diagnostic testing and early therapeutic intervention in symptomatic PIBD patients.

Current guidelines from ESPGHAN IBD Porto Group and ECCO indicate that opportunistic infections are more frequent in immunosuppressed IBD populations [6,7]. In our study, most non–*C. difficile* infections were caused by enteric pathogens commonly associated with pediatric gastroenteritis [10,20]. The generally mild course of these infections aligns with previous findings that, although IST increases susceptibility, the overall burden of severe bacterial infections in PIBD is low [6]. Nonetheless, the single case of septic CDI emphasizes the potential for serious outcomes in this population and reinforces the need for vigilance, particularly in patients with extensive colonic involvement or heightened inflammatory activity.

A central diagnostic challenge in PIBD lies in the considerable clinical overlap between infectious enteritis and disease flare. Symptoms such as diarrhea, abdominal pain, hematochezia, and elevated inflammatory markers may reflect active IBD, superimposed infection, or both [21,22]. Accordingly, the current ESPGHAN Porto Group position paper recommends diagnostic testing for *C. difficile* at initial PIBD diagnosis and in all patients with a suspected PIBD flare prior to treatment modification [23]. However, laboratory testing alone cannot reliably distinguish asymptomatic colonization from clinically relevant CDI, and clinical correlation, as well as toxin detection, is essential. Beyond CDI-focused testing during disease flares, guidance on broader and more systematic microbiological assessment in routine follow-up remains limited. Given that infection-associated flares in our cohort frequently led to IST escalation, optimized diagnostic strategies incorporating systematic pathogen evaluation in symptomatic patients may improve differentiation between inflammatory activity and infection. Further studies are needed to clarify whether expanded or more standardized microbial screening approaches could enhance clinical decision-making and long-term disease management in PIBD.

This study provides real-world insight into infection management in PIBD, an area for which explicit recommendations are currently limited. The frequent continuation or escalation of IST during active infection suggests that clinicians prioritize the risk of uncontrolled IBD over the theoretical risk of exacerbating infection, emphasizing the need for clearer PIBD-specific guidance on the management of immunosuppression during infectious episodes.

This study has several limitations. Its retrospective design, small sample size, and single-center setting limit generalizability and preclude inferential statistical analyses and robust assessment of risk factors associated with infection. In addition, the study focused exclusively on bacterial GI infections, and systematic screening for viral, fungal, or protozoal pathogens was not performed. Therefore, their potential contribution to PIBD flares cannot be excluded. Furthermore, data on patients with negative microbiological testing were not systematically collected, limiting comparative analyses.

Strengths of this study include the detailed, patient-level characterization of infection episodes, including microbiological findings, treatment strategies, and clinical outcomes over a 10-year period. Despite the limitations, the consistency of our findings with existing literature supports their clinical relevance.

## 5. Conclusions

In conclusion, bacterial GI infections in PIBD patients occurred at a rate of 3 per 100 person-years and were predominantly mild, with *C. difficile* being identified as the most common pathogen in this cohort. Infections frequently coincided with increased IBD activity, and IST was often continued or escalated, reflecting the clinical challenge of balancing disease control and infection risk. Although severe infections were rare, the occurrence of septic CDI illustrates that serious complications can occur. These findings support the importance of timely pathogen-specific diagnostics, careful clinical assessment, and the need for more specific guidance on immunosuppressive management during infectious episodes in PIBD.

By providing detailed, patient-level data on pathogen distribution, recurrence, immunosuppressive management, and antibiotic treatment strategies, this study contributes to the understanding of bacterial GI infections in PIBD and may help inform future PIBD-specific recommendations.

## Figures and Tables

**Table 1:** Patient characteristics, including PIBD type and phenotype, age at PIBD diagnosis and at infection, causative pathogen, and immunosuppressive and antibiotic treatment at the time of and during infection.

Patient /Episode	PIBD Diagnosis	Phenotype	Biological Sex	Age at IBD Diagnosis	Age at Infection	Pathogen	IST at Infection Onset	IST During Infection	ABT
P1	CD	ileitis, pancolitis, UGI	male	13	13	*C. difficile*	AZA	AZA	metronidazole
P2	UC	pancolitis	female	6	9	*C. difficile*	AZA	AZA, CS	metronidazole
P3	UC	pancolitis	female	12	13	*C. difficile*	CS	CS	metronidazole
P4	CD	ileitis, colitis, UGI	female	15	16	*C. difficile +* *S. Enteritidis*	none	MTX	none
P5/E1	CD	ileitis, colitis, UGI	female	16	17	*C. difficile*	AZA	AZA	metronidazole
P5/E2	CD	ileitis, colitis, UGI	female	16	17	*C. difficile*	AZA	AZA	none
P6	UC	pancolitis, UGI	male	10	12	*C. difficile*	CS	Increase in CS dose, AZA	none
P7	CD	ileitis, colitis, UGI	male	10	10	*K. oxytoca*	AZA	Increase in AZA dose, CS	ciprofloxacin
P8/E1	UC	pancolitis	female	14	14	*Aeromonas hydrophila*	none	none	amoxicillin
P8/E2	UC	pancolitis	female	14	16	*C. difficile*	TAC	TAC, AZA	cefotaxime, vancomycin
P9	UC	pancolitis	male	3	16	*Arcobacter butzleri*	none	none	none
P10	CD	colitis, UGI	male	17	18	*K. oxytoca*	MTX, IFX	MTX, IFX	metronidazole
P11	CD	colitis, UGI	male	8	18	*C. jejuni*	IFX	IFX	ceftriaxone, metronidazole
P12	CD	pancolitis, UGI	female	6	9	*C. difficile*	MTX, IFX	IFX	metronidazole (not taken)
P13/E1	CD	ileitis, colitis, UGI	female	13	14	*C. difficile*	MTX	MTX	none
P13/E2	CD	ileitis, colitis, UGI	female	13	14	*C. difficile*	MTX	MTX	none
P14	UC	colitis (only rectal biopsies) obtained), UGI	male	16	18	*C. difficile*	AZA, CS	AZA, increase in CS dose	metronidazole

Abbreviations: P—patient (P1–P14 refer to individual patients); E—episode (E1–E2 indicate first and second episode); PIBD—pediatric inflammatory bowel disease; UGI—upper gastrointestinal involvement; *C. difficile*—*Clostridioides difficile*; *S. Enteritidis*—*Salmonella Enteritidis*; *K. oxytoca*—*Klebsiella oxytoca*; *C. jejuni*—*Campylobacter jejuni*; IST—immunosuppressive therapy; AZA—azathioprine; CS—corticosteroids; TAC—tacrolimus; MTX—methotrexate; IFX—infliximab; ABT—antibiotic therapy.

**Table 2:** Laboratory findings in 14 PIBD patients with a total of 17 episodes of bacterial gastrointestinal infections.

Patient /Episode	WBC (G/L)	ANC (G/L)	HB (g/dL)	PT (G/L)	Albumin (g/dL)	CRP (mg/L)	ESR (mm, 1 h/2 h)	FCL (µg/g)	FOB	Pathogen
P1	11.66	7.9	12.3	431	3.3	80.1	55/80	2521	n.a.	*C. difficile*
P2	5.42	n.a.	12.2	448	4	5.1	n.a.	3211	neg.	*C. difficile*
P3	13.7	n.a.	9.2	749	2.8	50.7	36/75	883	n.a.	*C. difficile*
P4	11.7	n.a.	12.6	176	n.a.	15.2	n.a.	655	neg.	*C. difficile +* *S. Enteritidis*
P5/E1	5.89	3.7	11.8	470	3.6	60.3	66/109	n.a.	n.a.	*C. difficile*
P5/E2	6.18	4.3	11.5	499	3.9	43.9	60/112	>1800	neg.	*C. difficile*
P6	14.34	10.6	14.3	339	4.5	1.9	n.a.	118	n.a.	*C. difficile*
P7	10.97	8	10.7	580	2.6	171.3	n.a.	>1000	n.a.	*K. oxytoca*
P8/E1	6.46	n.a.	8	454	4.5	3.3	n.a.	>1800	n.a.	*Aeromonas* *hydrophila*
P8/E2	14.17	9.3	11.4	455	3.9	65	n.a.	n.a.	pos.	*C. difficile*
P9	5.46	2.8	16.3	265	4.8	<0.6	3/10	19	neg.	*Arcobacter* *butzleri*
P10	13.8	10.6	15.2	288	n.a.	9	n.a.	n.a.	n.a.	*K. oxytoca*
P11	10.83	6.4	16.1	335	4.4	55.5	n.a.	942	neg.	*C. jejuni*
P12	9.89	5.8	12.3	381	3.4	18.4	n.a.	2427	n.a.	*C. difficile*
P13/E1	11.17	8.3	14.3	235	4.1	3.9	n.a.	1232	n.a.	*C. difficile*
P13/E2	12.38	9.3	13.4	236	n.a.	7.3	n.a.	>1800	n.a.	*C. difficile*
P14	21.9	n.a.	11.2	473	n.a.	21.6	28/82	>1000	n.a.	*C. difficile*

Abbreviations: PIBD—pediatric inflammatory bowel disease; P—patient (P1–P14 refer to individual patients); E—episode (E1–E2 indicate first and second episode); n.a.—not available; WBC—white blood cell count; G/L—10^9^ cells per liter; ANC—absolute neutrophil count; HB—hemoglobin; g/dL—grams per deciliter; PT—platelet count; Albumin—serum albumin; CRP—C-reactive protein; mg/L—milligrams per liter; ESR—erythrocyte sedimentation rate; mm, 1 h/2 h—mm/h, 1 and 2 h measurements; FCL—fecal calprotectin; µg/g—micrograms per gram; FOB—fecal occult blood; *C. difficile*—*Clostridioides difficile*; *S. Enteritidis*—*Salmonella Enteritidis*; *K. oxytoca*—*Klebsiella oxytoca*; *C. jejuni*—*Campylobacter jejuni*.

## Data Availability

The data presented in this study are not publicly available due to privacy and ethical restrictions. De-identified data may be made available by the corresponding author upon reasonable request and with approval of the Ethics Committee of the Medical University of Graz.

## Abbreviations

The following abbreviations are used in this manuscript:

AB	Antibiotics
ABT	Antibiotic therapy
Albumin	Serum albumin
ANC	Absolute neutrophil count
AZA	Azathioprine
CBC	Complete blood count
CD	Crohn’s disease
*C. difficile*	*Clostridioides difficile*
*C. jejuni*	*Campylobacter jejuni*
CRP	C-reactive protein
CS	Corticosteroids
E	Episode (E1–E2 indicate first and second episode)
ESR	Erythrocyte sedimentation rate
FCL	Fecal calprotectin
FOB	Fecal occult blood
GI	Gastrointestinal
G/L	10^9^ cells per liter
g/dL	Grams per deciliter
HB	Hemoglobin
IFX	Infliximab
IBD	Inflammatory bowel disease
IST	Immunosuppressive therapy
*K. oxytoca*	*Klebsiella oxytoca*
MEDOCS	Medical documentation system (hospital information system)
mg/L	Milligrams per liter
mm, 1 h/2 h	mm/h, 1 and 2 hour measurements
MTX	Methotrexate
P	Patient (P1–P14 refer to individual patients)
PICU	Pediatric intensive care unit
PIBD	Pediatric inflammatory bowel disease
PT	Platelet count
PCT	Procalcitonin
*S. Enteritidis*	*Salmonella Enteritidis*
TAC	Tacrolimus
UGI	Upper gastrointestinal tract involvement
UC	Ulcerative colitis
WBC	White blood cell count
µg/g	Micrograms per gram
